# Olfactory Dysfunction and Cognitive Deterioration in Long COVID: Pathomechanisms and Clinical Implications in Development of Alzheimer’s Disease

**DOI:** 10.3390/cells15020176

**Published:** 2026-01-19

**Authors:** Egidio Stigliano, Aurora Tocci, Rita Florio, Vincenzo Arena, Giuseppina Amadoro

**Affiliations:** 1 Anatomic Pathology Unit, Department of Woman and Child Health and Public Health, Fondazione Policlinico Universitario Agostino Gemelli IRCCS, Largo Francesco Vito 1, 00168 Rome, Italy; vincenzo.arena@unicatt.it; 2 Anatomic Pathology Unit, Ospedale Isola Tiberina-Gemelli Isola, Via di Ponte Quattro Capi 39, 00186 Rome, Italy; aurora.tocci@fbf-isola.it; 3 European Brain Research Institute (EBRI), Viale Regina Elena 295, 00161 Rome, Italy; r.florio@ebri.it; 4 Institute of Translational Pharmacology (IFT), National Research Council (CNR), Via Fosso del Cavaliere 100, 00133 Rome, Italy

**Keywords:** Severe Acute Respiratory Syndrome CoronaVirus 2 (SARS-CoV-2), COronaVIrus Disease of 2019 (COVID-19) syndrome, anosmia, olfactory bulbs (OBs), Alzheimer’s Disease (AD), cognitive impairment

## Abstract

**Highlights:**

**What are the main findings?**

- Neurological complications, including loss of smell, cognitive and psychiatric symptoms, contribute to long COVID syndrome.
- A correlation exists between persistent anosmia and clinical dementia in patients who experienced SARS-CoV-2 infection.

**What are the implications of the main findings?**

- SARS-CoV-2 infection of olfactory neuroepithelium may contribute to degeneration of limbic and cortical brain areas and, then, to the onset of Alzheimer’s Disease (AD).
- Interventions for treatment of SARS-CoV-2-mediated chronic olfactory dysfunction are required to improve brain and mental health in COVID-19 survivors.

**Abstract:**

Complete or partial loss of smell (anosmia), sometimes in association with distorted olfactory perceptions (parosmia), is a common neurological symptom affecting nearly 60% of patients suffering from post-acute neurological sequelae of COronaVIrus Disease of 2019 (COVID-19) syndrome, called long COVID. Severe Acute Respiratory Syndrome CoronaVirus 2 (SARS-CoV-2) may gain access from the nasal cavity to the brain (neurotropism), and the olfactory route has been proposed as a peripheral site of virus entry. COVID-19 is a risk factor for developing Alzheimer’s Disease (AD), an age-dependent and progressive neurodegenerative disorder characterized in affected patients by early olfaction dysfunction that precedes signs of cognitive decline associated with neurodegeneration in vulnerable brain regions of their limbic system. Here, we summarize the recent literature data supporting the causal correlation between the persistent olfactory deterioration following SARS-CoV-2 infection and the long-delayed manifestation of AD-like memory impairment. SARS-CoV-2 infection of the olfactory neuroepithelium is likely to trigger a pattern of detrimental events that, directly and/or indirectly, affect the anatomically interconnected hippocampal and cortical areas, thus resulting in tardive clinical dementia. We also delineate future advancement on pharmacological and rehabilitative treatments to improve the olfactory dysfunction in patients recovering even from the acute/mild phase of COVID-19. Collectively, the present review aims at highlighting the physiopathological nexus between COVID-19 anosmia and post-pandemic mental health to favor the development of best-targeted and more effective therapeutic strategies in the fight against the long-term neurological complications associated with SARS-CoV-2 infection.

## 1. Pathomechanisms of Olfactory Dysfunction in Coronavirus Disease 2019 (COVID-19) Syndrome

SARS-CoV-2 is the etiological agent of the multi-organ COronaVIrus Disease of 2019 (COVID-19) syndrome whose pandemic outbreak was in late 2019 in Wuhan, China. After the World Health Organization (WHO) declared the COVID-19 pandemic a public health emergency in January 2020, there have been over 7 million deaths worldwide more than two years ago (https://www.who.int/emergencies/diseases/novel-coronavirus-2019/situation-reports, accessed on 1 November 2023). The most relevant clinical features of COVID-19 are fever (98.6%), fatigue (69.6%), dry cough (59.4%), myalgia (34.8%), and dyspnea (31.2%), whereas less common symptoms include headache (6.5%), dizziness (9.4%), abdominal pain (2.2%), diarrhea (10.1%), nausea (10.1%), and vomiting (3.6%) [1]. Some patients may present a rapid progression to a life-threatening complication named Acute Respiratory Distress Syndrome (ARDS), which affects 3.4% of infected patients and 15.6–17.0% of severe cases [1]. A proportion of individuals diagnosed with COVID-19 (about 10%) presents a variety of long-lasting symptoms that negatively affect their quality of life, including debilitating neurological and neuropsychiatric complications, anxiety (16%), hyposmia–hypogeusia (20%), encephalitis (13%), cognitive impairment or “brain fog” (14%) and peripheral neuropathies (25%). This condition, enduring for at least six months after the acute infection, was initially called Post COVID-19 Condition (PCC) or post-acute sequelae of COVID-19 (PASC) but is more commonly termed long COVID [2,3,4].

Consistent with this chain of events, a growing body of clinical and experimental evidence has shown that SARS-CoV-2 is not merely a pulmonary pathogen being endowed with neuroinvasive properties and neural pathogenicity [5,6,7,8,9,10,11]. Although a growing body of evidence has shown that neuroinvasion is extremely rare in humans [6], SARS-CoV-2 may enter the brain via the hematogenous route and peripheral nerves, including axonal anterograde transport via the olfactory nerve (CNI) [12,13,14,15,16,17,18,19]. Viral particles can be actively transported via motor proteins (kinesin and dynein) along microtubules of the neuroanatomy network and/or diffuse passively to penetrate the Central Nervous System (CNS), thus resulting in injury of neural and glial structures along with severe neuroinflammatory responses, cytokine storm and thrombotic microangiopathy [20]. Additionally, compelling investigations have largely documented Olfactory Bulbs (OBs) damage and a sequential impaired sense of smell (anosmia) following nasal infection with the SARS-CoV-2 virus, with a prevalence ranging from 5 to 98.3% depending on areas, populations, SARS-CoV-2 variants, and diagnostic methodologies [21,22,23,24]. In support of the virus neurotropism, several studies carried out in both humans and animal models have reported that SARS-CoV-2 virus is able to directly infect neurons and glial cells in the CNS [25,26,27,28,29]. Consistently, the expression of Angiotensin-Converting Enzyme II (ACE2)—the functional host receptor for SARS-CoV-2 entry—has been found in astrocytes from rat cerebellum and brainstem [30,31], in human middle temporal gyrus and posterior cingulate cortex [32], in cranial nerves (glossopharyngeal and vagal nerves) [33], and in mitral cells of mouse OBs [34,35]. In agreement, SARS-CoV-2 readily targets neurons of ACE2-expressing 3D human brain organoids, giving rise to tau hyperphosphorylation and mislocalization into soma, DNA fragmentation, and, eventually, cell death [36]. Moreover, in a human ACE2 knockin mouse generated by using CRISPR/Cas9 technology, intranasal inoculation of SARS-CoV-2 virus leads to marked brain deterioration in concomitance with robust viral S protein expression in neurons, astrocytes, and microglial cells [37]. However, despite evidence produced using multiple experimental approaches, differences in sensitivity of methodology used (immunohistochemistry, Real-Time Quantitative Reverse Transcription Polymerase Chain Reaction (qRT-PCR); single-cell gene expression analysis), collection and preservation protocols of human samples (CSF, autoptic specimens, whole brain, and selected cerebral area), sample size and model organism (transgenic, organoid, and cell lines) have raised inconsistencies that cast doubts on the actual neurovirulence of different SARS-CoV-2 strains. Furthermore, other plausible pathophysiological mechanisms, including vasculopathy and hypercoagulability [38,39], Blood Brain Barrier (BBB) leakage, deficits in brain perfusion/oxygenation and hypoxia exacerbating the pro-inflammatory state and massive cytokine release [40], encephalitits and meningoencephalitis (these conditions are extremely rare in COVID syndrome) [41] might be all causative factors in the alterations of neuronal circuits (demyelination, reduction in synaptic connections, and neuronal survival) following SARS-CoV-2 infection [42,43], highlighting that the actual nature of the neurological symptoms associated with COVID-19 syndrome is more complicated than that ascribed to direct viral invasion of the brain. As a consequence, even though the olfactory pathway has been initially proposed as main route of brain infection of SARS-CoV-2, the evidence of frank viral infection of the Olfactory System (OS), particularly of resident neurons, is still lacking up to now [44,45,46,47,48,49], and losing olfaction in COVID-19 is supposed to have more likely a multifactorial cellular basis [22,50].

In this regard, compelling clinical and experimental evidence has shown that neuronal infection is extremely rare in the OS [51], hinting at the possibility that the olfactory injury and correlated odor dysfunction are not direct consequences of local, virus-induced, neuronal damage [24,27,44,45,51]. First, the two obligatory gateway receptors ACE2 and TransMembrane PRoteaSe Serine type 2 (TMPRSS2) required for the Spike protein priming and virus fusion with the host cell membrane are not expressed by a large part of Olfactory Sensory Neurons (OSNs) that are not permissive and, then, only rarely infected by SARS-CoV-2 virus [43,46,52,53,54,55,56,57]. Proof-of-concept data of single-cell sequencing followed by immunohistochemistry validation on human nasal biopsies have clearly revealed that both ACE2 and TMPRSS2 are mainly expressed by the SUStentacular cells (SUSs), mucus-secreting Bowman’s gland cells, stem cells including Globose and Horizontal Basal cells (GBCs and HBCs, respectively), and vascular pericytes rather than by OSNs that do express only the two co-receptors NeuRoPilin-1 (NRP1) and BaSiGinor or emmprin (BSG, CD147) involved in facilitation of the virus infectivity [52,58,59]. Likewise, no signs of viral protein are detected in OSNs, whereas the nucleocapsid of SARS-CoV-2 is contextually discernible in SUSs of the human olfactory mucosa [51]. The SARS-CoV-2 N nucleoprotein is also visualized in SUSs and, only to a lesser extent, in mature and immature OSNs both in Syrian hamsters and human specimens [60,61,62]. Reactive gliosis with hypertrophy of astrocytes and microglia is clearly visible from OBs specimens of deceased subjects with confirmed diagnosis of COVID-19 syndrome in the absence of frank signs of viral infection (RNA and particles) and measurable expression level of ACE2 receptor despite strong positivity in their corresponding lungs [63,64]. Notably, by taking advantage of novel and minimally-invasive technique with the Jamshidi needle as a transethmoidal probe, with low risk of contamination for healthcare personnel and the autopsy room, Stigliano et al. [63,64] confirmed that OSNs are not the main cellular target of SARS-CoV-2 infection and replication. Second, the timing in regeneration of receptor OSNs and in virus trafficking via anterograde axonal transport through the olfactory nerve (CNI) and transynaptic migration is not compatible with the abrupt onset of complete anosmia and its recovery [44,45,65]. Third, the virus localization within OSNs is not unequivocal, with several false positives due to dissimilarities in experimental procedures (immunocytochemistry, in situ hybridization, electron microscopy, RT-PCR), tools (antibodies and non-physiological animal models), and tissue sampling (biopsy, nasal brushing, and autopsy collection). Another layer of complexity is added by the lack of an objective measure of OS function and its poor clinical correlation with corresponding biological findings [21,23,51,66,67]. Fourth, SARS-CoV-2 does not invade the olfactory nerves in patients with COVID-19, and these nerve endings remain intact during anosmia [51,68]. Fifth, the number of OSNs is not significantly decreased following SARS-CoV-2, indicating that these receptor-expressing neurons in COVID-19 patients with anosmia are impaired by different mechanisms than viral infection [45,68]. Sixth, viral mechanisms of SARS-CoV-2 trafficking (nervus terminalis or cranial nerve 0) as well, beyond the olfactory nerve that traverses the cribriform plate, are possible routes to gain access to the brain [25,44,48,49,69,70].

On the basis of the literature data that do not support beyond question the involvement of the direct SARS-CoV-2 infection of OSNs as responsible for olfactory damage [23,46,69,71], several alternative, non-mutually exclusive neuropathogenic events have been proposed to be causally associated with the disablement of smell identification in COVID-19 affected patients. First, SARS-CoV-2 infection of the SUSs—providing structural support and energy (glucose) and facilitating the molecular movement of airborne odor molecules—and/or of the basal cells (BCs)—involved in homeostatic regeneration during the normal turnover of olfactory epithelial and upon tissue destruction [50,52]—leads indirectly to impairment of OSNs and, then, associated anosmia [47,72]. Infection of OSNs might arise from horizontal virus spread from the neighboring supporting cells or from dissemination of the virus into the olfactory epithelium due to disarrangement of its architecture owing to inflammatory infiltration [68,73]. Second, SARS-CoV-2-induced insult of vascular endothelial cells triggers a neuroimmune response (“cytokine storm”) with consequent exaggerated release of cytokines and other harmful proinflammatory mediators—such as InterLeukin-6 (IL-6), Tumor Necrosis Factor alpha (TNF-α), InterFeroN-gamma (IFN-γ) and CXC chemokine ligand 10 (CXCL10)—causing a secondary damage to nasal epithelial cells and related olfactory function by means of changes in gene expression for odorant receptors (and additional signaling molecules), deciliation (reduction of motile dendritic cilia) and/or immune response injury [62,74,75,76,77,78]. Third, anosmia is also consistent with the virus-mediated secretion of small molecules/peptides affecting the Choline AcetylTransferase (ChAT)-expressing neurons located in the OBs and receiving axons from the magnocellular nuclei of the basal forebrain of cortical areas [79]. Alternatively, the nasal cavity obstruction, oedema of the olfactory cleft mucosa, and mucus reduction that impedes the diffusion and signal transduction activated by odorants might be the culprit of OS failure in COVID-19-affected patients as well [73].

Taken together, these findings argue against the direct viral invasion with damage to OSNs (neurotropism) and successive neuropathic disarrangement [51,80,81], suggesting a possible non-cell-autonomous pathomechanism(s) of elimination of supporting cells and host immune response as the main factor responsible for anosmia during COVID-19 infection. Despite the absence of ACE2 and TMPSSR expression in OSNs, it’s more conceivable that SARS-CoV-2 invasion both harms the non-neuronal cells located in the olfactory neuroepithelium (SUSs and BCs) and provokes an aggravating inflammatory response [72,82], ensuing in impairment of resident neuronal cells and stem cells, prolonged odour deterioration, and smell deprivation [34]. Persistent hyposmia (weakened sense of smell), parosmia (aberrant sense of smell), and anosmia (complete and irreversible loss of smell) are also related to abnormal tissue regeneration and protracted immune response [46,71].

Of note, the mechanisms of acute (lasting around 2–3 weeks) and chronic (persisting and potentially permanent) olfactory dysfunction with consequent loss of smell significantly differ from each other. Initially, a reorganization of Olfactory Epithelium (OE) cytoarchitecture occurs, followed by a more sustained inflammatory state with chronic release of cytokine [83]. In detail, at the beginning, inflammation impairs the OSNs and up-regulates the HBC-mediated regeneration, but the prolonged inflammatory state converts over time the HBCs into an undifferentiated state, which is characterized by huge expression of NF-κB-regulated cytokines and chemokines, including CCL19, CCL20, and CXCL10 [84]. Reduction in the number of OSNs, with infiltration of IFNγ-producing T cells and dendritic cells, along with depletion of M2 macrophages, also contribute to long-term anosmia in COVID-19 patients [77]. Viral and host risk factors, including genetic variation, age, gender, and racial ethnicity, can further contribute to the phenotypic spectrum of anosmia, in particular for patients suffering long COVID [77,85,86].

## 2. Persistent Anosmia After SARS-CoV-2 Infection Is Associated with an Increased Long-Term Risk of Developing Cognitive Impairment Related to Age-Related Neurodegenerative Alzheimer’s Disease (AD)

Although the biological bases and molecular pathways underlying anosmia following exposure to SARS-CoV-2 are still a matter of debate in the literature published [87], several lines of evidence indicate that post-COVID-19 olfactory dysfunction might be a prodromal marker and/or an early readout for a clinical–pathological trajectory leading to neurodegeneration [88,89]. In this framework, prolonged and/or relapsing alterations of OBs in anosmic patients might have an early and prognostic value for the majority of affected patients in predicting an extensive neurodegeneration associated with neuropsychological symptomatology of poor mnemonic and emotional performance, consistent with long COVID symptomatology [90,91,92,93].

**Figure 1:**
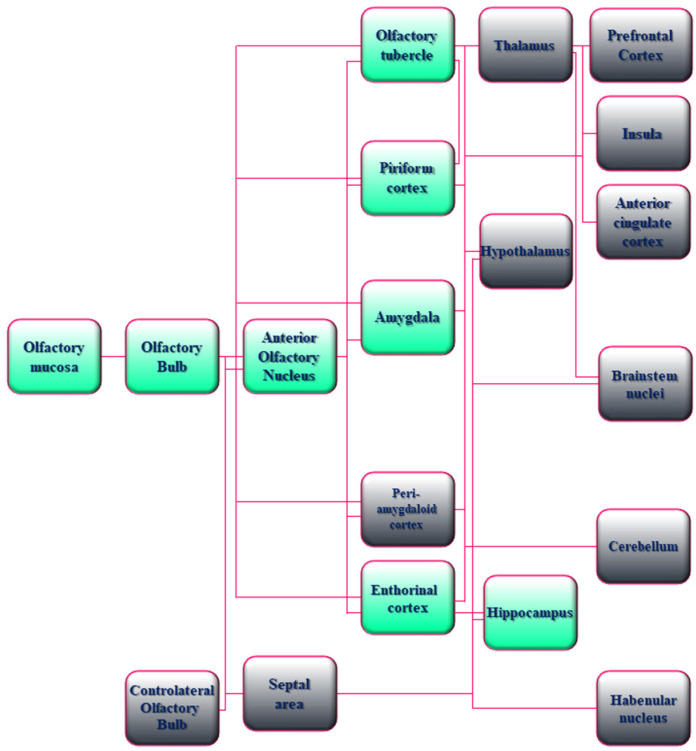
Schematic drawing of the anatomical and functional organization of the Olfactory System (OS) in humans. A flowchart of the hierarchical organization of the olfactory network in humans is shown. Boxes with green shading indicate the AD-relevant olfactory and limbic system connectivity. Boxes with gray shading indicate the other sensory, neuromodulatory, and motor olfactory cortical structures. In detail, the neuro-epithelium located in the olfactory mucosa of the human nasal cavity consists of bipolar OSNs whose axons coalesce to form the olfactory nerves that transmit processing signals by making excitatory synapses (glomeruli) onto the apical dendrites of Mitral and Tufted cells of OBs. The binding of airborne odorant molecules to specific receptors located on cilia of OSNs triggers a G-coupling protein-mediated intracellular transduction signaling with resultant generation of a Cyclic-Adenosine MonoPhosphate (cAMP) second messenger, opening of the cyclic nucleotide-gated ion channels, and an influx of calcium and sodium ions, along with membrane depolarization and action potentials. The process of detection and specific information transmission requires multiple permutations between odor molecules and receptors because a single odor receptor can be activated by numerous similar odorants and vice versa. The OBs receiving inputs from the periphery project directly to the Anterior Olfactory Nucleus (AON) into the primary olfactory cortex and other contiguous regions with diverse associated functions including emotional processing of olfactory stimuli (AmyGdala (AG) and adjacent PeriAmygdaloid Cortex (PAC)), odor-induced motivated behavior (Olfactory Tubercle (OT)), odor-context episodic and working memory (entorhinal cortex–hippocampus), odor quality coding and discrimination (Piriform Cortex (PC)). Neuronal connections of these regions are reciprocal and bidirectional, allowing the integration of associative information about smell at the level of the primary olfactory cortex. The secondary olfactory cortex consists of structures (THalamus (TH), HypoThalamus (HT), HiPoCampus (HPC), and the portion of the PreFrontal Cortex (PFC) named the OrbitoFrontal Cortex (OFC)) receiving direct projections from the primary olfactory cortex. The nuclei of the TH send connections towards the orbitofrontal/insular/cingulate cortices and the motor areas of the brainstem involved in high-order functions such as odor-evoked reward, modulation of odor perception, and behavioral-guided emotional responses. Cerebellar (CB) and habenular (Hb) nuclei receiving projections from primary olfactory neurons, even though they do not directly participate in olfactory perception, play a role in smell-related motor performance.

From an anatomic point of view, OBs are integral parts of the limbic system, including olfactory, amygdala, and hippocampal regions that have been referred for a long time as “rhinencephalon” or “smell brain” [94,95]. In particular, the human OS requires a hierarchical organization to convey information about the smell from the nasal epithelium to the primary and secondary olfactory cortices of the brain. First, the olfactory nerve—the bundle of axons (fila olfactoria) from sensory receptor neurons devoted to detecting odor molecules within the nasal epithelium—penetrates the small foramina in the cribriform plate of the ethmoid bone and transmits excitatory signals to OBs that represent the first cerebral relay station of olfactory neurocircuitry. Upon entry into the cranial cavity, these fibers converge and make synaptic boutons named “glomeruli” on the output neuronal population called “Tufted and Mitral cells”, which are located in the External Plexiform Layer (EPL) and Mitral Cell Layer (MCL), respectively. From the glomeruli, processed information throughout second and third order nerves are then passed into the olfactory tract to upper cortical and subcortical regions, such as Anterior Olfactory Nucleus (AON), Piriform Cortex (PC), Cortical Amygdaloid nucleus (CoA) and Lateral Entorhinal Cortex (LEC) (primary olfactory cortex) and, in turn, to the PreFrontal Cortex (PFC), the OrbitoFrontal Cortex (OFC) and the HiPpoCampus (HPC) (secondary olfactory cortex) [96,97,98]. Noteworthy is that these regions dealing with the sense of smell are also critical for emotion, motivation, and long-term memory [95] (Figure 1). From a physiological point of view, both in rodent experimental models and in humans, OBs are functionally connected to the limbic system by sending projections and receiving inputs from Lateral Entorhinal Cortex (LEC), ventral HiPpoCampus (vHPC), and AmyGdala (AG) across a sensory–limbic neuronal network [98,99,100]. Therefore, a close anatomical and functional coupling exists between the odor-sensing olfactory areas and the high-order limbic regions, which are essential for cognitive processing and are selectively vulnerable in AD neurodegeneration. In accordance, comprehensive studies have underscored that olfactory bulbectomy and/or disruption of olfactory circuits in experimental animal models provoke several neurochemical, anatomical, and behavioural abnormalities typically associated with a classification of AD phenotype. For instance, impairment in memory and learning functions, disturbances in long-term synaptic plasticity, depletion of cholinergic neurons in the medial septum–diagonal band in combination with decrease of acetylcholine content and acetylcholinesterase, reduction in dendritic arborization and spine density, changes in neurogenesis, accumulation of extra-cellular Amyloid beta (Aβ) and hyperphosphorylation of intraneuronal tau protein with deposition of insoluble aggregated inclusions, hippocampal and neocortical atrophy are all downstream events connected with cytopathic insult of OS [101,102,103,104,105,106,107,108,109,110,111,112]. Besides, hyposmia is tightly linked with neuropathological brain changes of olfactory–limbic system connections at prodromal stages of AD before any detectable sign of cognitive impairments [113]. Furthermore, even though a direct, causal relationship between chronic smell impairment and increased susceptibility to AD has not yet been definitively demonstrated, an association between cognition and olfaction has been recently documented in long COVID [67,88,114]. More specifically, it has been proposed that SARS-CoV-2 infections in anosmic subjects might trigger and/or accelerate pathological progression of presymptomatic neurodegeneration [24,115,116,117]. In particular, a significant reduction in grey matter thickness in the OFC and ParaHippocampal Gyrus (PHG) regions supporting memory and neuropsychiatric function has been reported in association with an increment in tissue injury markers in primary olfactory cortex (temporal PC and AON) and cognitive decline by a longitudinal study carried out on 785 participants of UK Biobank, including 401 cases tested positive for infection with SARS-CoV-2 and underwent Magnetic Resonance Imaging (MRI) scans with 141 days separating their initial diagnosis and the second imaging analysis [116].

On this point, a positive correlation between anosmia and cognitive and memory disturbances due to temporo-mesial dysfunction is reported in another study enrolling 18 patients with post-COVID-19 syndrome that underwent neuropsychological assessment and evaluation of clinical parameters approximately 2–3 months after recovery [118]. Likewise, in a cohort of 66 participants with acute or persistent COVID-19 anosmia, smell deterioration turns out to be linked with poor cognitive functions (short-term memory, working memory, visuospatial abilities, and orientation) in objective tests, adding more support to the occurrence of a clear relationship between the chronicity of olfactory disablement and memory complaints [119]. Persistent anosmia is significantly correlated with behavioural, functional and structural brain alterations (reduction in functional activity during the making-decision task, thinning of cortico-parietal regions, and dissipation in white matter integrity) in a large cohort of patients recovering from COVID-19, strongly supporting the fact that the persistent loss of smell is a reliable proxy biomarker for long-term brain and cognitive impairment following SARS-CoV-2 infection [120]. An increased vulnerability to verbal memory breakdown, accompanied by changes in delta-band EEG connectivity and White Matter Hyperintensity (WMH) volumes are reported in a large cohort of COVID-19 survivors with acute hyposmia at 10 months after infection resolution [121]. Moreover, another study reports moderate deficit in prefrontal-related cognitive responses in post-COVID-19 subjects with chronic hyposmia up to 4 months after the end of the infection, as assessed by means of a comprehensive battery of neuropsychological and functional Near-Infrared Spectroscopy-ElectroEncephaloGram (fNIRS-EEG) co-recording techniques and Sniffin’ Sticks test paradigms aimed at testing their cognitive performance in concomitance with parameters of cerebral hemodynamic/bioelectrical activity and smell abilities [122]. In a similar way, a positive association between the reduction in integrity of the olfactory network, hyposmia severity, and neuropsychological performance in visuospatial memory and executive functions is observed in 23 patients with persistent (11 months and more) COVID-19-related anosmia in comparison to their sex- and age-matched controls [123]. Once more, mental clouding is reported in 71 (46.7%) out of 152 long COVID patients that had suffered from olfactory impairment and anosmia over 6 months from infection and underwent olfactometry, nasal endoscopy, headache scale, cognitive assessment, proving that a chronic, recrudescent smell deficit following exposure to SARS-CoV-2 greatly increases the probability of future neurodegenerative disease, including memory disorders [114]. On the contrary, no changes in amyloid-β peptide, tau protein, Neurofilament Light chain (NfL) and other AD markers have been detected in peripheral olfactory neurons from individuals with persistent (for 6 to 10 months) post-COVID-19 hyposmia when compared to age/sex-matched healthy controls, indicating that the loss of smell due to SARS-CoV-2 infection is not likely to predispose to future neurodegeneration [124]. Besides, an increased level of oxidative and neuroinflammatory stress in correlation with alteration in olfaction-associated genes has been found in a 3D in vitro model of olfactory mucosa from demented individuals with AD when compared to sister control cultures upon exposure to SARS-CoV-2, corroborating the existence of pathological cross-talk between COVID-19 and neurodegeneration [125]. Once more, post-acute COVID-19 survivors with olfactory defects complain of worse memory performance or “brain fog” in comparison to those without hyposmia/anosmia when objectively evaluated by means of both self-report surveys and clinical assessments of cognition in Montreal Cognitive Assessment (MoCA) and Mini-Mental State Examination (MMSE) tasks [126,127,128,129,130,131,132]. On the contrary, a significant trend between the recurrent olfaction and memory deterioration after the acute phase of COVID-19 has not been found in other prospective, longitudinal, case-control studies [133,134,135,136]. In particular, in one investigation performed on 141 confirmed cases of COVID-19 with prolonged neurological symptoms (more than 3 months), a lower frequency of anosmia is even detected in patients with cognitive impairment in comparison to the healthy and Subjective Cognitive Decline (SCD) groups, although prolonged hospitalization in the intensive care unit, cerebral ischemia and hypoxemia might have been confounding factors [137]. Finally, in 168 participants hospitalized for mild to moderate COVID-19 and evaluated at 6 months after discharge, the prevalence of objective hyposmia and subtle cognitive deficits is higher in older subjects [138], in line with results from a previous population-based investigation [139].

It’s important to emphasize that contradictory findings in the COVID-19 research are in part due to the urgency of the pandemic outbreak, with consequent heterogeneity in studies carried out [140]. Conflicting results can be ascribed to discrepancies in: (i) the study design (sample size, endpoints and observation periods, virus variants with diverse olfactory prevalence and disease severity, information registries about the participants’ mental health at baseline, and data collection of vaccination status); (ii) the methodologies used to assess the olfactory dysfunction and cognitive status (differing screening methods, diagnostic criteria and subjective (self-report) or objective outcome measures); and (iii) the low level of control for pre-existent confounders (risk factors and comorbidities) and non-standardized investigation of post-infection complications. Additional limitations are the lack of distinction in the COVID-19 group between participants with hyposmia and normosmia and between survivors from the early, more severe, and subsequent waves of SARS-CoV-2 infection. Alternatively, the occurrence of compensatory response of the neural network following temporary or permanent loss of smell should also be taken into account for results interpretation and comparability [141].

## 3. How Can SARS-CoV-2 Infection Promote the Onset of Alzheimer’s Disease?

Given that an intrinsic association between odour identification and spatial memory exists in humans regardless of the incidence of diseased states, the key question that arises in this field is what may be the possible cause(s) of the development of neurodegeneration and dementia in post-acute COVID-19 survivors with smell loss [88,142,143].

To start with, although most cases of COVID-19 patients in clinical practice recover from the loss of olfaction within 8 weeks from the initial infection and in highly variable way, some of them retain protracted and sometimes permanent olfactory deficits [77,144], likely due to immune cell infiltration and gene expression alteration of neuroepithelium residing in the nasal cavity, including OSNs [49]. Olfactory detriment, or lower ability to identify odors, is a preclinical sign of AD patients [145,146] by predicting the manifestation of early/moderate pathological stages associated with a reduction in cognitive scores and brain atrophy occurring in selected limbic structures, especially the entorhinal cortex and hippocampus [147,148,149,150,151]. Individuals with amnestic Mild Cognitive Impairment (MCI) and olfactory deficits have a higher risk of their illness evolving into full-blown AD dementia with cerebral burden of Aβ, as shown in 63 participants undergoing olfactory identification task and Positron Emission Tomography (PET) imaging using Pittsburgh Compound B (PiB) [152,153]. Relevantly, the build-up of Aβ and tau into aggregated inclusions, named Senile Plaques (SPs) and NeuroFibrillary Tangles (NFTs), reactive gliosis with activation of inflammatory signalings, neuritic dystrophy, global decrease in gray matter, production of deleterious free radicals, neurotransmitter (cholinergic, serotonergic, and noradrenergic) alterations, cellular senescence and neuronal death, are all pathognomonic hallmarks of AD taking place at multiple levels of the peripheral and central OS of affected subjects (olfactory epithelium, OBs/olfactory tract, olfactory nucleus and cortices) and mirroring similar neuropathology into their memory and cognitive-related higher brain centers, such as hippocampus and cerebral cortex [23,113,154,155,156,157,158]. In addition, COVID-19 survivors, particularly elderly individuals, display an increased susceptibility to developing AD-like memory complaints in response to severe outcomes, and vice versa, AD subjects manifest a greater vulnerability to SARS-CoV-2 infection, thus indicating that an epidemiological connection exists between these two complex and multifaceted syndromes that share neuropathological convergences, common risk factors, and mechanistic aspects [85,159,160]. In agreement, is the evidence that SARS-CoV-2 infection causes in the nasal neuroepithelium the release of few pro-inflammatory cytokines, chemokines, in particular IL-1β and IL-6 and TNFα, and the activation of transcription factors, such as the NF-κB/NLRP3 inflammasome, that correlate with the severity of hyposmia after COVID-19 and overlap with the analogous intracellular signal transduction pathways and immune mediators related to AD neurodegeneration [161,162,163]. In addition, the dysregulation of immune response occurring in the brain in response to SARS-CoV-2 infection triggers a marked oxidative stress with production of Reactive Oxygen Species (ROS) and NitrOgen Species (NOS) that further contribute in vicious feed-forward cycle to aggregation of insoluble Aβ fibrils and propagation of neuronal damage [164,165]. Interestingly, infection of the nasal epithelium by neurotropic human viruses, for instance Herpes Simplex Virus (HSV, Types 1 and 2) and Herpesvirus (Types 6A and 6B), elicits an exaggerated neuroinflammatory response in the brain that, in turn, promotes the production of Aβ known to be endowed with natural antimicrobial protective property [166].

Additionally, just as other viral pathogens [167,168], SARS-CoV-2 infection (which shows low neurotropic property in humans [51]) directly and/or indirectly leads, per se, to a boost in cerebral amyloidosis by accelerating in mice the Amyloid Precursor Protein (APP)/Aβ metabolism because of an upregulation of β/γ secretase processing complex and/or an interference with its intracellular trafficking and clearance and/or a promotion of its seeding property [169,170,171]. Further complicating the phenomenon at hand, Aβ deposition triggers downstream several adverse consequences (tau hyperphosphorylation, oxidative stress, ionic imbalance, proteostasis, aberrant membrane receptor stimulation, disruption of neuronal network (excitatory–inhibitory imbalance), alteration in synaptic transmission and plasticity, DNA/RNA injury, axonopathy, apoptosis), causing cytotoxic insult to the surrounding parenchyma and ending up in neuronal shrinkage and loss [172]. Consistently, an increase in tau hyperphosphorylation at several AD-relevant epitopes with mislocalization into the somatic compartment and high propensity to form insoluble aggregates are observed following the exposure to SARS-CoV-2 in human SH-SY5Y neuron-like cell line and transgenic mouse expressing human ACE2 (K18-hACE2), an animal model that does not completely reflect the human pathophysiology [173]. Of note, in a mouse model overexpressing a humanized mutant form of APP (hAPP) into OSNs, a progressive spreading of Aβ through the OS towards the hippocampus is detected with aging [174], consistent with the “olfactory vector hypothesis” in which xenobiotics and toxins are supposed to trigger the prion-like transneuronal propagation of amyloid proteopathic seeds from the nose to brain during AD development [175,176,177]. Furthermore, the Spike glycoprotein of SARS-CoV-2 interacts and interferes with α7 nicotinic AcetylCholine Receptors (nAChR) [178,179] that are present both in the nose and in the basal forebrain, corroborating that a hypofunction/disruption of the cholinergic system together with inflammatory response and redox imbalance occur both in COVID-19 and AD. Relevantly, the adenovirus-mediated expression of SARS-CoV-2 S1 in nasal cavity of mice is proved to cause marked OBs impairment due to a local increment of apoptotic markers associated with diminution of intracerebral acetylcholine production and hippocampal neurogenesis, excessive neuroinflammation and neurological complications that are mitigated by administration with donepezil, an U.S. Food and Drug Administration (FDA)-approved cholinergic compound used clinically for treatment of moderate or severe cognitive disturbances in AD patients [180].

Moreover, MRI and cognitive longitudinal analyses have revealed in a large cohort of participants of UK Biobank diagnosed positive for infection with SARS-CoV-2 that a greater alteration in markers of tissue damage occurs into their limbic structures in association with decline in memory performance, likely due to extensive neuroinflammation, disease spread throughout the olfactory pathways and/or loss of sensory input with anosmia [181]. An abnormal build-up of AD-related tau protein hyperphosphorylation accompanied by sustained glia stimulation and upregulation in inflammatory factors have been detected in the hippocampus and medial entorhinal cortex from postmortem human brain samples within 4–13 months of recovery from acute COVID-19 [182]. More recently, in a prospective and case-control study performed on 36 hyposmic and 26 normosmic participants who had experienced mild COVID-19 compared to 25 healthy controls, an atrophy in the OBs and thickness of related cortical structures (left orbital sulci) have been detected in correlation with lower cognitive performance especially in language skills in the relatively long-term period (402  ±  215.8 days), just as in elderly individuals and people with AD [183]. Notably, the majority of the above-mentioned studies are correlational with follow-up periods of less than 12 months, which are not long enough to accurately predict (and fully recapitulate) the entire neuropathological and clinical spectrum of AD. As a consequence, additional longitudinal investigations with prolonged observation time points (more than 1 year) aimed at assessing the functional and cognitive consequences of structural brain changes occurring in COVID19 survivors are still required to prove a direct, causal involvement of SARS-CoV 2 infection for the alterations of limbic brain via the olfactory route in triggering the full-blown AD phenotype.

**Figure 2:**
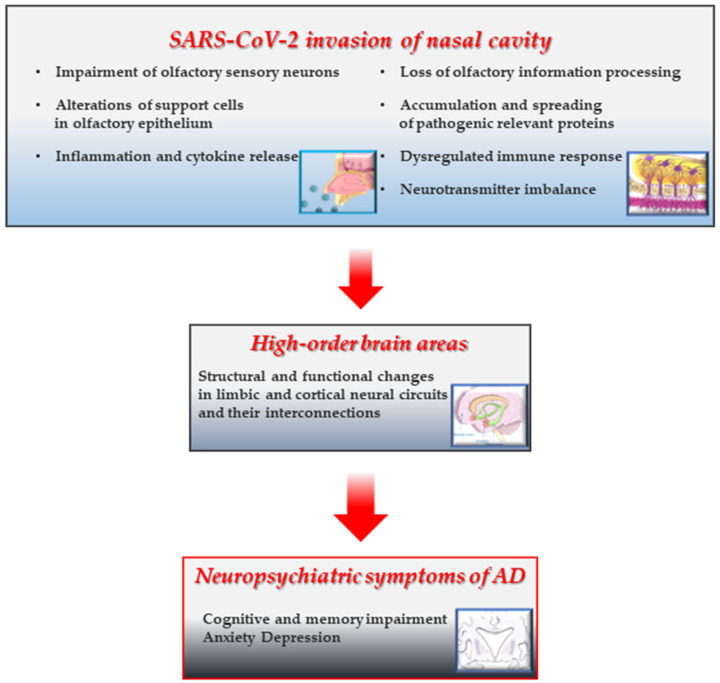
SARS-CoV-2 infection of the nasal neuroepithelium may trigger a cascade of AD-like damaging events leading to degeneration of the limbic system involved in cognitive memory. A graphic illustrating the possible pathomechanisms underlying the neurodegenerative alterations of the limbic system and related AD-like amnestic and neuropsychiatric symptoms following nasal infection of SARS-CoV-2 in COVID-19 subjects with persistent anosmia. The presence of SARS-CoV-2 in the nasal cavity and/or the long-term maladaptive, self-perpetuating responses following its acute invasion locally cause the disruption of cytoarchitecture and integrity of neuroepithelium (reduction in regeneration, sensory deciliation, deprivation of energy supply, and gene expression alteration) [51,52,60,65,186], persistent inflammatory infiltration) [17,62,77], proteostasis with accumulation/aggregation/spreading of toxic proteins [173,174], dysregulation in neurotransmission and network connectivity [178,179]. These insults trigger (and/or accelerate) long-range noxious effects on anatomically-interconnected higher limbic (and cortical) centers [115,116,120,121,122,123,181,182,183] whose progressive deterioration is associated with episodic memory and cognition decline in AD pathology. Prolonged or dysregulated responses following olfactory infection of SARS-CoV-2 could initiate transition to AD-like neuropathology [114,118,119,129,130,131,183,184]. This figure was partly generated using Servier Medical Art (https://smart.servier.com, accessed on 15 October 2025) licensed under a Creative Commons Attribution 3.0 unported license (https://creativecommons.org/licenses/by/3.0/, accessed on 15 October 2025).

Despite this limitation and in light of the current studies cited in the present review, it has been suggested that: (i) the presence of olfactory impairment in patients recovering from COVID-19 might be a predictor of dementia-like decline in middle-aged and older adults [184]; (ii) anosmia and cognitive deterioration may arise from both direct and indirect effects (or combination of them) of SARS-CoV-2 nasal infection that may instigate and/or precipitate the underlying neuropathological processes linked with development of AD and clinical dementia [88,116,171,184,185] (Figure 2). From the OB, the virus might potentially gain access to different regions within the neuraxis (neuroinvasion), but, as previously reported, SARS-CoV-2 does not appear to be a neurotropic virus in humans [186]. In addition to infection of support cells (SUS and Bowman gland cells), followed by the deciliation and disruption of OE integrity [51], a deleterious effect on the OE resulting in inflammation and, then, in neuronal impairment is considered to be another plausible mechanism (i.e., immune reaction of the host) by which SARS-CoV-2 causes anosmia [27]. To this end, previous studies published before the COVID-19 pandemic outbreak have clearly shown that persistent nasal inflammation caused by deleterious environmental agents, including viruses, induces in mice a loss of OSNs and then atrophy of the OB [187]. In detail, the damage is due to the shrinkage of superficial layers of the OB (i.e., the Olfactory Nerve Layer (ONL), Glomerular Layer (GL), and EPL). The shrinkage of the ONL and GL originates from the retraction of OSNs axons (and loss of synapses) and gross injury of the Olfactory Epithelium (OE) owing to nasal inflammation [188,189]. Likewise, COVID-19 patients with anosmia display imaging and neuropathological findings of neuroinflammation and OB atrophy with changes in higher olfactory structures, such as reduction in gray matter mass and hypometabolism assessed by PET scans [190]. Relevantly, an association exists between nasal inflammation and neurologic disorders, including AD [191], supporting the notion that an indirect impairment of OSNs due to neuroinflammation with subsequent OB atrophy might affect the downstream synaptically-interconnected limbic circuits. Consistently, a marked inflammatory and degenerative pathology (microglial activation with neurophagia) in the absence of signs of viral RNA or protein (except for nasal epithelium) has been reported into the brains of a large cohort of COVID-19 cases from Columbia University Irving Medical Center/New York Presbyterian Hospital, indicating that direct SARS-CoV-2 infection of brain tissues does not account for the neuropathological changes [192].

Another conceivable pathogenesis of COVID-19-induced anosmia may involve the disuse of the olfactory sense (i.e., odor deprivation). In this context, several studies have shown that loss of smell in adults in response to an insult (acquired anosmia) triggers structural and functional changes in upper cortical areas associated with olfactory processing [193,194]. In line, olfactory impairment has been reported to be associated with signs of neurodegeneration such as lower volume in the fusiform gyrus and the middle temporal cortex (including the hippocampus and entorhinal cortex) and accelerated AD-like cognitive decline over 15 years [195]. Apart from the convergence of common molecules and biological pathways, the evidence that the SubGranular Zone (SGZ) of the hippocampus and the SubVentricular Zone (SVZ) of OBs are both sites of adult neurogenesis may also account for the selective vulnerability of these two neuronal circuitries and the coupled anosmia/AD-like dementia phenotypic manifestation in COVID-19. Attenuations in hippocampal and olfactory neurogenesis due to impairment of endogenous neural stem cell activity might be the neuropathological substrate linking limbic neurodegeneration and hyposmia/anosmia following SARS-CoV-2 infection. Impairments in olfaction and cognition occurring in COVID-19 syndrome may arise from the SARS-CoV-2 virus-mediated alteration of physiological neurogenesis that is known to play key roles in maintenance/regeneration of olfaction and cognitive learning/memory processes [196].

## 4. Therapy for Treatment of Olfactory Impairment in COVID-19

Understanding the intricate and variegated nature of the OBs damage following the SARS-CoV-2 infection and its mechanistic aspects is of paramount importance to design effective therapeutic interventions and long-term preventive strategies to counteract and/or mitigate the potential neurological complications in affected patients with long COVID sequelae. Notably, recent observations have shown that the incidence of olfactory deterioration is greatly variable among different SARS-CoV-2 genetic variants, calling into question the different outcomes and prognosis of individuals with chronic anosmia and/or parosmia after the initial disease presentation. Historically, the first wave of COVID-19 emerged in China in 2019 (the original Wuhan D614 strain (20A.EU2)) and spread to the rest of Asia, Europe, the Middle East, Australia, and North and South America. The spread was accompanied by the appearance of the G614 virus mutation, which was much more efficient in infecting the olfactory epithelium, resulting in greater olfactory dysfunction than the original D614 variant. Since the diffusion of the G614 virus throughout the world, several new SARS-CoV-2 virus variants have emerged—Alpha (B.1.1.7), Beta (B.1.351), Gamma (B1.1.28), and Delta (B.1.617.2)—that carry the D614G mutation and are similarly harmful to olfaction [197]. In 2022, the Omicron variant (B.1.1.529) emerged, which has numerous mutations in its Spike protein compared with the original strain of SARS-CoV-2 and is less neuroinvasive with scarse propensity to infect the olfactory epithelium owing to its strong hydrophobicity, alkalinity and lower solubility into the mucus in comparison to Delta (B.1.617.2) or Alpha (B.1.1.7) ones [198,199]. In keeping with this finding, the prevalence of long COVID with neurological complications might be lower in individuals infected by the Omicron variant than those infected by other variants [200]. Besides, a possible genetic predisposition to COVID-19-related anosmia has been proposed by a multi-ancestry Genome-Wide Association Study (GWAS) reporting in individuals, especially of European ancestry, who experienced loss of smell following SARS-CoV-2 infection due to the presence of UDP Glucuronosyltransferase Family 2 Member A1 and A2 (UGT2A1 and UGT2A2), two genes that are expressed in the olfactory epithelium and involved in metabolizing odorants and detoxifying compounds [201]. Additional risk factors for olfactory impairment in COVID-19 patients are proposed to be smoking, lifestyle, allergic conditions, sex, age, body size, race, physical inactivity, hypertension, heavy alcohol consumption and diabetes mellitus causing differences in cytokine production profile, relative abundance of neurons and glial cells in the olfactory epithelium, different expression level of ACE2 and TMPRSS2 in SUSs, microvascular and inflammatory components [202]. Although different studies have provided conflicting results about what are actually the predisposing factors for persistent COVID-19-induced olfactory impairment (for details see [23,202]), smoking, history of allergy (particularly respiratory), and Type 2 Diabetes Mellitus (T2DM) appear to significantly increase the risk for smell loss in COVID-19 patients [203,204].

Rehabilitation and/or treatments with drugs possessing potent antioxidant, anti-inflammatory, neuroregenerative, modulatory, and neuroprotective properties are currently used, sometimes in a combined regimen, to relieve the post-viral COVID-19 olfactory dysfunction. In this framework, olfactory training (OT) [205,206] based on the repeated inhalation exposure of four odorant agents (clove, citronella, eucalyptus, and phenylethyl alcohol) together with supplement-based oral therapy and pharmacological (local or systemic) interventions, including vitamin A or its metabolite Retinoic Acid (RA) [207,208], omega-3 fatty acid [209], zinc sulphate trace element [210], Alpha-Lipoic Acid (ALA) [211], PalmitoylEthanolamide fatty Acid (PEA) with flavonoid Luteolin (PEA-LUT) [212], gabapentin [213,214], Cerebrolysin [215], 13-cis-retinoic acid plus aerosolized Vitamin D [216], dexamethasone [217], corticosteroids [218,219,220], insulin [221,222], autologous Platelet-Rich Plasma enriched with growth factors (PRP) [223,224], buffer solutions (sodium citrate, tetra sodium pyrophosphate and sodium gluconate) [225,226,227] are under investigation in recent prospective trials. Some agents (for all details, including number of clinical trials, phase distribution and a proposed management algorithm for clinicians see [21,228,229]) requires additional testing (omega-3 fatty acid supplement, gabapentin, insulin, PRP, vitamin A are in Phase 2/3) while others appear more effective being at the end of their clinical experimentation (aerosolized all trans-retinoic acid plus Vitamin D, cerebrolysin, dexamethasone, corticosteroids nasal irrigation and PEA-LUT are in Phase 4). In particular, the most promising drugs appear to be PEA, a bioactive lipid mediator with anti-inflammatory and neuroprotective activities, administered in combination with LUT, a flavonoid (3,4,5,7-tetrahydroxy flavone) contained in plants with anti-inflammatory and anti-oxidant properties, and Cerebrolysin, a neuropeptide preparation with neuroprotective and neurotrophic actions similar to those of neurotrophic growth factors [228,229,230]. Transplantation of Mesenchymal Stem Cells (MSCs), implantation of an electrical device for stimulation of the OBs, and intranasal delivery of small interfering RNAs (siRNAs) for SARS-CoV-2 RNA hold promise as innovative therapeutic options, but they still require further validation. Standardization of protocols and outcome assessments, along with long-term studies are needed in the future to compare the safety, feasibility, and effectiveness of different central and peripheral-acting therapeutic options for chronic COVID-19-associated smell loss over placebo, even though a personalized treatment might be desirable due to differences in individual susceptibility and SARS-CoV-2 variants [229,230].

## 5. Conclusions

Evidence is accumulating on the pathological connection between long COVID following SARS-CoV-2 infection and neurodegeneration [231]. The olfactory neural circuit is an entry route of virus neurotropism to the brain, and anosmia is an early symptom of patients suffering from COVID-19. Several commonalities and underlying mechanisms have been described in support of the risk factors between COVID-19 and the initial stages of AD development [85]. The main purpose of the present review is to provide evidence that hyposmia due to COVID-19 infection causes modifications in the OB and olfactory-related cortical structures in the CNS, but whether these changes in the brain may univocally predict over time the cognitive decline remains to be elucidated. To date, the knowledge of the long-term impact of SARS-CoV-2 on the CNS is yet unknown due to the insufficient time elapsed since the COVID19 pandemic outbreak. It has been proposed that effects of SARS-CoV-2 infection propagated throughout the olfactory network may accelerate brain aging and favor the later onset/development of age-related AD-like neurodegeneration and dementia even years after the acute phase of viral infection. The virus invasion of the sinonasal cavity may directly spread, likely through exosomes, to the higher-order neuroanatomically-connected limbic and cortical areas involved in cognition and episodic memory, and/or initiate a local chain of degenerative events (dysregulated immune response, misfolding/aggregation of toxic proteins) that, in turn, affect the neuron function/survival and glial reactivity. As an alternative, virus-triggered systemic cytokine storm and increased BBB permeability indirectly amplify and/or promote neurodegenerative processes of these deep temporal nuclei associated with sensory inputs [171,232]. The selection of reliable SARS-CoV-2 animal models that approximate the human disease process of long-term neurodegeneration is urgently required for studying the etiology and testing the therapeutic efficacy of vaccines or drugs in pre-clinical studies. In addition, longitudinal and cross-sectional and case-control studies assessing pairwise cognitive and olfactory performances in conjunction with classical biomarkers (Aβ 1–42, phosphorylated tau, and NfL) in CerebroSpinal Fluid (CSF) or plasma from patients recovering from neurological sequelae of acute COVID-19 are needed in the near future to understand whether the chronic anosmia with olfactory deterioration actually contributes to the onset and/or worsening AD manifestation or is merely a bystander epiphenomenon. A combination of pharmacological, nutritional, and rehabilitative approaches is also recommended to re-establish the olfactory epithelial homeostasis in the management of post-COVID-19 olfactory dysfunction and its psychological, neuropsychiatric, and cognitive consequences.

## Data Availability

No new data were created or analyzed in this study.

## Abbreviations

COronaVIrus Disease of 2019 (COVID-19) syndrome; Severe Acute Respiratory Syndrome CoronaVirus 2 (SARS-CoV-2); Alzheimer’s Disease (AD), Acute Respiratory Distress Syndrome (ARDS); Central Nervous System (CNS); Angiotensin-Converting Enzyme II (ACE2); TransMembrane PRoteaSe Serine type 2 (TMPRSS2); Olfactory Sensory Neurons (OSNs); SUStentacular cells (SUSs); Globose and Horizontal Basal Cells (GBCs and HBCs); NeuRoPilin-1 (NRP1); BaSiGinor (BSG); Olfactory System (OS); Olfactory Bulbs (OBs); Anterior Olfactory Nucleus (AON); AmyGdala (AG); PeriAmygdaloid Cortex (PAC); Olfactory Tubercle (OT); Piriform Cortex (PC); THalamus (TH); HypoThalamus (HT); HiPoCampus (HPC); PreFrontal Cortex (PFC); OrbitoFrontal Cortex (OFC); InterLeukin-6 (IL-6); Tumor Necrosis Factor alpha (TNF-α); InterFeroN-gamma (IFN-γ) and CXC chemokine ligand 10 (CXCL10); Montreal Cognitive Assessment (MoCA); Mini-Mental State Examination (MMSE); Subjective Cognitive Decline (SCD); CerebroSpinal Fluid (CSF); small interfering RNAs (siRNAs); Reactive Oxygen Species (ROS); NitrOgen Species (NOS); Amyloid Precursor Protein (APP); Amyloid beta (Aβ); Genome-Wide Association Study (GWAS); UDP Glucuronosyltransferase Family 2 Member A1 and A2 (UGT2A1 and UGT2A2); SubGranular Zone (SGZ); SubVentricular Zone (SVZ); Mild Cognitive Impairment (MCI); Positron Emission Tomography (PET); Pittsburgh Compound B (PiB); Senile Plaques (SPs); NeuroFibrillary Tangles (NFTs); Blood Brain Barrier (BBB); Retinoic Acid (RA); Alpha-Lipoic Acid (ALA); PalmitoylEthanolamide fatty Acid (PEA); Mesenchymal Stem Cells (MSCs); Cerebellum (CB); Habenula (Hb).
